# Polarity and Ferromagnetism in Two-Dimensional Hybrid Copper Perovskites with Chlorinated Aromatic Spacers

**DOI:** 10.1021/acs.chemmater.2c00107

**Published:** 2022-02-21

**Authors:** Ceng Han, Alasdair J. Bradford, Jason A. McNulty, Weiguo Zhang, P. Shiv Halasyamani, Alexandra M. Z. Slawin, Finlay D. Morrison, Stephen L. Lee, Philip Lightfoot

**Affiliations:** † School of Chemistry and EaStChem, University of St Andrews, St Andrews KY16 9ST, United Kingdom; ‡ School of Physics, University of St Andrews, St Andrews, Fife KY16 9SS, United Kingdom; § Department of Chemistry, University of Houston, Houston, Texas 77204, United States

## Abstract

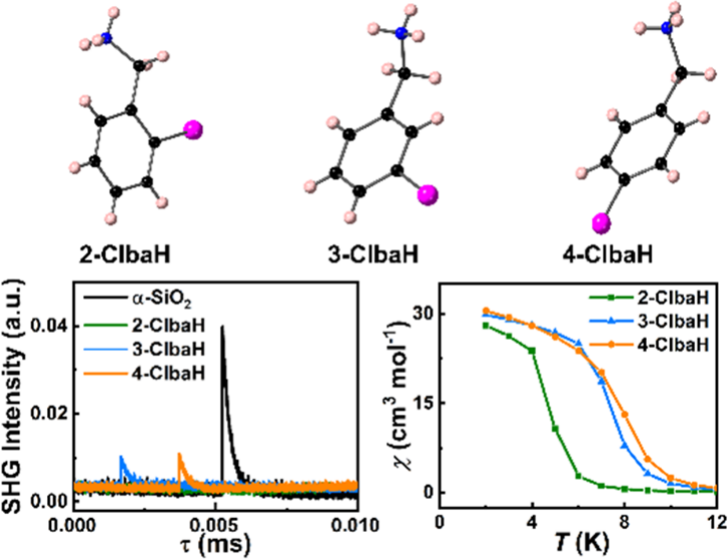

Two-dimensional (2D) organic–inorganic hybrid copper halide perovskites have drawn tremendous attention as promising multifunctional materials. Herein, by incorporating *ortho*-, *meta*-, and *para*-chlorine substitutions in the benzylamine structure, we first report the influence of positional isomerism on the crystal structures of chlorobenzylammonium copper(II) chloride perovskites A_2_CuCl_4_. 2D polar ferromagnets (3-ClbaH)_2_CuCl_4_ and (4-ClbaH)_2_CuCl_4_ (ClbaH^+^ = chlorobenzylammonium) are successfully obtained. They both adopt a polar monoclinic space group *Cc* at room temperature, displaying significant differences in crystal structures. In contrast, (2-ClbaH)_2_CuCl_4_ adopts a centrosymmetric space group **P**2_1_/**c** at room temperature. This associated structural evolution successfully enhances the physical properties of the two polar compounds with high thermal stability, discernible second harmonic generation (SHG) signals, ferromagnetism, and narrow optical band gaps. These findings demonstrate that the introduction of chlorine atoms into the interlayer organic species is a powerful tool to tune crystal symmetries and physical properties, and this inspires further exploration of designing high-performance multifunctional copper-based materials.

## Introduction

Organic–inorganic hybrid halide perovskites have recently received tremendous interest owing to their extraordinary photovoltaic and optoelectronic properties.^1−3^ Among them, two-dimensional (2D) hybrid perovskites can be regarded as derived by slicing the three-dimensional (3D) cubic perovskite aristotype ABX_3_ along vertices of the BX_6_ octahedra and inserting additional organic moieties between these layers.^4^ Among the known families of 2D layered perovskites, two conventional families are the Dion–Jacobson (DJ)^5^ and Ruddlesden–Popper (RP)^6^ phases, which are commonly defined in terms of their generic stoichiometries ABX_4_ and A_2_BX_4_, respectively, for examples with single octahedral layers. The tolerance factor constraint that occurs in 3D perovskites can be considerably relaxed in 2D hybrid perovskites, endowing them with striking structural flexibility and novel functionalities.^7−9^ As a result, the crystal structures and physical properties of 2D hybrid perovskites can be modulated by much larger and more complex organic molecules with various sizes and functional groups.^10−12^ A halogen substitution strategy, especially introducing fluorine atoms into the organic molecules, has been proved to be a powerful approach to enhance the ferroelectric performance in 2D lead-based halide perovskites.^13,14^

Copper-based halide perovskites have recently been studied intensively for developing new multifunctional materials because of their interesting thermochromism, ferromagnetism, and ferroelectricity.^15−17^ The Cu-based perovskites possess the inherent benefits of lower toxicity and greater light and humidity stability, in comparison to Pb-, Cd-, Sn-, or Bi-based perovskites.^18,19^ Structurally, the Jahn–Teller (J–T) distortion of the 3d^9^ ion Cu^2+^ results in the elongation of octahedral coordination, introducing additional flexibility into copper-based systems.^20^ Consequently, more complex organic moieties can be included in copper halide perovskites to adjust the physical and chemical properties. To date, a few copper-based halide perovskites with halogen-substituted organic molecule spacers have been prepared. The introduction of a single fluorine atom at the *meta*-position in the benzylamine structure leads to a polar ferromagnet (3-FbaH)_2_CuCl_4_ (3-FbaH^+^ = 3-fluorobenzylammonium).^21^ A multiaxial ferroelectric (DF-CBA)_2_CuCl_4_ (DF-CBA = 3,3-difluorocyclobutylammonium) with a ferroelectric phase transition temperature of 380 K is successfully obtained by incorporating two fluorine substituents in the centrosymmetric (CBA)_2_CuCl_4_ (CBA = cyclobutylammonium) structure.^22^ Heavier halogen substitutions, such as chlorine or bromine substitution in ethylamine, influence the reversible and irreversible thermochromism in 2D layered perovskites (CEA)_2_CuCl_4_ (CEA = 2-chloroethylammonium) and (BEA)_2_CuCl_4_ (BEA = 2-bromoethylammonium).^23^

However, there is no report on multifunctional 2D copper-layered perovskites by introducing chlorine atoms into isomeric organic molecules at various positions. Herein, for the first time, we report the influence of positional isomerism on the crystal structures of chlorobenzylammonium copper(II) chloride perovskites A_2_CuCl_4_ by incorporating *ortho*-, *meta*-, and *para*-chlorine substitutions in the benzylamine structure. The monochlorine substitution at various positions in the benzylamine structure changes the crystal symmetries and physical properties. We present 2D polar ferromagnets (3-ClbaH)_2_CuCl_4_ (3-ClbaH^+^ = 3-chlorobenzylammonium) and (4-ClbaH)_2_CuCl_4_ (4-ClbaH^+^ = 4-chlorobenzylammonium). They both crystallize in a polar monoclinic space group *Cc* at room temperature, displaying significant differences in crystal structures. However, their isomeric analogue (2-ClbaH)_2_CuCl_4_ (2-ClbaH^+^ = 2-chlorobenzylammonium) crystallizes in a centrosymmetric space group **P**2_1_/**c** at room temperature. This associated structural evolution successfully enhances the physical properties of the two polar compounds, which exhibit high thermal stability, discernible second harmonic generation (SHG) signals, polar-to-nonpolar phase transition temperatures up to 433 K, ferromagnetism, and narrow optical band gaps.

## Experimental Section

### Materials

Anhydrous copper(II) chloride (CuCl_2_, 98%), hydrochloric acid (HCl, 36%, w/w aqueous solution), and absolute ethanol (C_2_H_5_OH, 99.99%) were purchased from Alfa Aesar. 2-Chlorobenzylamine (C_7_H_8_NCl, 95%), 3-chlorobenzylamine (C_7_H_8_NCl, 98%), and 4-chlorobenzylamine (C_7_H_8_NCl, 98%) were purchased from Fluorochem. All chemicals were directly used without further purification.

### Synthesis

The compounds (2-ClbaH)_2_CuCl_4_, (3-ClbaH)_2_CuCl_4_, and (4-ClbaH)_2_CuCl_4_ were crystallized by a slow evaporation method.

For (2-ClbaH)_2_CuCl_4_ (C_14_H_18_N_2_CuCl_6_), CuCl_2_ (268.9 mg, 2 mmol) was dissolved in concentrated HCl (5 mL) with moderate heating. 2-Chlorobenzylamine (0.5 mL, 4 mmol) was added. Once fully dissolved, the solution was allowed to cool. By cooling for several hours, green, platelet-shaped crystals were obtained. Elemental anal. calcd (%) for (2-ClbaH)_2_CuCl_4_: C, 34.28; H, 3.70; N, 5.71. Found: C, 34.58; H, 3.56; N, 5.52.

For (3-ClbaH)_2_CuCl_4_ (C_14_H_18_N_2_CuCl_6_), CuCl_2_ (134.45 mg, 1 mmol) was dissolved in concentrated HCl (20 mL) with slow stirring and moderate heating. Once fully dissolved, 3-chlorobenzylamine (0.24 mL, 2 mmol) was added. The produced precipitates were dissolved by adding excess concentrated HCl and ethanol to get a clear solution. By naturally cooling the solvent for a few hours, yellow, platelet-shaped crystals were obtained. Elemental anal. calcd (%) for (3-ClbaH)_2_CuCl_4_: C, 34.28; H, 3.70; N, 5.71. Found: C, 34.55; H, 3.52; N, 5.54.

For (4-ClbaH)_2_CuCl_4_ (C_14_H_18_N_2_CuCl_6_), CuCl_2_ (134.45 mg, 1 mmol) was dissolved in concentrated HCl (20 mL) with moderate heating. Once fully dissolved, 4-chlorobenzylamine (0.24 mL, 2 mmol) was added. The produced precipitates were dissolved by adding excess concentrated HCl and ethanol to get a clear solution. By cooling for a few hours, yellow, platelet-shaped crystals were obtained. Elemental anal. calcd (%) for (4-ClbaH)_2_CuCl_4_: C, 34.28; H, 3.70; N, 5.71. Found: C, 34.53; H, 3.49; N, 5.54.

## Characterization

### Single-Crystal X-ray Diffraction

Single-crystal X-ray diffraction data were collected on a Rigaku SCX Mini diffractometer at 173 and 298 K using Mo Kα radiation (λ = 0.71075 Å). The data were processed using Rigaku *CrystalClear* software.^24^ Absorption corrections were conducted empirically from equivalent reflections according to multiscans using *CrystalClear*.^24^ Crystal structures were solved using structure solution program *SHELXT*,^25^ and full-matrix least-squares refinements on *F*^2^ were carried out using *SHELXL-2018*/*3*^25^ incorporated in the WinGX program.^26^ All of the hydrogen atoms were treated as riding atoms, and all non-H atoms were refined anisotropically.

### Powder X-ray Diffraction (PXRD)

Powder X-ray diffraction data were measured on a PANalytical EMPYREAN diffractometer using Cu K_α1_ (λ = 1.5406 Å) radiation at ambient temperature. The data were collected in the range of 3–70° for 1 h to confirm the phase purity of each sample.

### Thermogravimetric Analyses (TGA)

TGA data were collected on an STA-780 instrument between 293 and 523 K at a heating rate of 5 K min^–1^ under a flowing N_2_.

### Second Harmonic Generation (SHG) Measurements

Samples were filled in fused silica tubes with an outer diameter of 4 mm. Relevant comparisons with known SHG material, α-SiO_2_, were made at the same condition. A 1064 nm pulsed Nd:YAG laser (Quantel Laser, Ultra 50) generated the fundamental light, and the SHG intensity was recorded at room temperature on an oscilloscope (Tektronix, TDS3032).

### Dielectric Measurements

Dielectric measurements were made on pellets ca. 1 mm thick and 10 mm in diameter formed by uniaxially pressing powder under a load of 2 tonnes. Silver conductive paste was applied to the opposing pellet faces and allowed to dry at 373 K. The data were recorded over the frequency range 100 Hz and 10 MHz at a heating/cooling rate of 3 K min^–1^ for (2-ClbaH)_2_CuCl_4_ and 1 K min^–1^ for (3-ClbaH)_2_CuCl_4_ and (4-ClbaH)_2_CuCl_4_ with the furnace working temperature between 298 and 470 K.

### Magnetic Measurements

The magnetic measurements were carried out on a Quantum Design (MPMS XL) SQUID magnetometer. Data were collected by cooling a known mass of material at 100 Oe field between 300 and 2 K. Measurements of magnetization versus applied field were carried out between −5000 and 5000 Oe at different temperatures.

### UV–Vis Absorption Spectral Measurements

Ambient temperature solid UV–vis absorbance spectra of powder samples were collected on a JASCO-V550 ultraviolet–visible spectrophotometer with a wavelength range of 200–900 nm.

## Results and Discussion

### Crystal Structures

The single-crystal X-ray structures suggest no phase transitions in the regime 173 < *T* < 298 K, so the crystallographic details will be discussed based on the structures at 298 K. Details of the structures at 173 K are provided in Supporting Information (SI). Crystallographic parameters for all three compounds at 298 K are given in Table 1, and selected geometrical parameters are given in Table 2.

**Table 1:** Crystal and Refinement Data for (2-ClbaH)_2_CuCl_4_, (3-ClbaH)_2_CuCl_4_, and (4-ClbaH)_2_CuCl_4_ at 298 K

compound	(2-ClbaH)_2_CuCl_4_	(3-ClbaH)_2_CuCl_4_	(4-ClbaH)_2_CuCl_4_
formula	C_14_H_18_N_2_CuCl_6_	C_14_H_18_N_2_CuCl_6_	C_14_H_18_N_2_CuCl_6_
formula weight	490.54	490.54	490.54
color/habit	green/platelet	yellow/platelet	yellow/platelet
crystal size (mm^3^)	0.27 × 0.18 × 0.05	0.25 × 0.15 × 0.05	0.50 × 0.50 × 0.02
crystal system	monoclinic	monoclinic	monoclinic
space group	**P**2_1_/**c**	*Cc*	*Cc*
*a* (Å)	17.0101(13)	10.4321(7)	33.656(3)
*b* (Å)	7.1189(5)	10.8034(8)	5.2632(4)
*c* (Å)	8.1375(5)	33.825(2)	10.5961(8)
α (deg)	90	90	90
β (deg)	102.228(4)	98.743(3)	98.883(6)
γ (deg)	90	90	90
*V* (Å^3^)	963.04(12)	3763.6(4)	1854.5(3)
*Z*	2	8	4
ρ_calcd_ (g cm^–3^)	1.692	1.731	1.757
μ (mm^–1^)	1.964	2.011	2.040
*F*(000)	494	1976	988
no. of reflns collected	9395	18558	7335
independent reflns	2198	8431	3229
	[*R*(int) = 0.1378]	[*R*(int) = 0.0666]	[*R*(int) = 0.0422]
goodness of fit	1.125	1.019	0.923
final *R* indices (*I* > 2σ(*I*))	*R*_1_ = 0.0653	*R*_1_ = 0.0588	*R*_1_ = 0.0263
	w**R**_2_ = 0.1794	w**R**_2_ = 0.1415	w*R*_2_ = 0.0531
largest diff. peak/hole (e Å^–3^)	0.904/–0.629	0.588/–0.665	0.258/–0.276

**Table 2:** Cu–Cl Bond Lengths and Cu–Cl–Cu Bond Angles for the Three Structures at 298 K

compound	(2-ClbaH)_2_CuCl_4_	(3-ClbaH)_2_CuCl_4_		(4-ClbaH)_2_CuCl_4_
	Cu1	Cu1	Cu2	Cu1
*R*_S_ (Å)	2.318(4)	2.320(4)	2.295(5)	2.295(3)
		2.324(4)	2.272(5)	2.301(3)
*R*_L_ (Å)	3.238(4)	3.036(5)	3.056(5)	2.993(3)
		2.904(5)	3.149(5)	3.009(3)
*R*_Z_ (Å)	2.262(5)	2.285(5)	2.285(5)	2.280(2)
		2.290(5)	2.296(5)	2.284(2)
[*R*_L_ – (*R*_S_ + *R*_Z_)/2] (Å)^29^	1.095	0.768	0.942	0.821
Cu–Cl–Cu (deg)	155.7(6)	167.9(4)–169.6(4)		174.1(3)–176.4(3)

The powder X-ray diffraction (PXRD) patterns of (2-ClbaH)_2_CuCl_4_, (3-ClbaH)_2_CuCl_4_, and (4-ClbaH)_2_CuCl_4_ all show similar characteristics to those calculated from their single-crystal structures (Figure S1). The phase purity of the powder samples was confirmed by elemental analysis and supported by Rietveld refinements (Figure S2), although a strong preferred orientation effect is observed due to the platelet morphology of the crystals. Thermogravimetric analysis (TGA) data reveal that these compounds show good thermal stability, with similar onset decomposition temperatures reaching 440, 443, and 453 K for (2-ClbaH)_2_CuCl_4_, (3-ClbaH)_2_CuCl_4_, and (4-ClbaH)_2_CuCl_4_, respectively (Figure S3).

**Figure 1:**
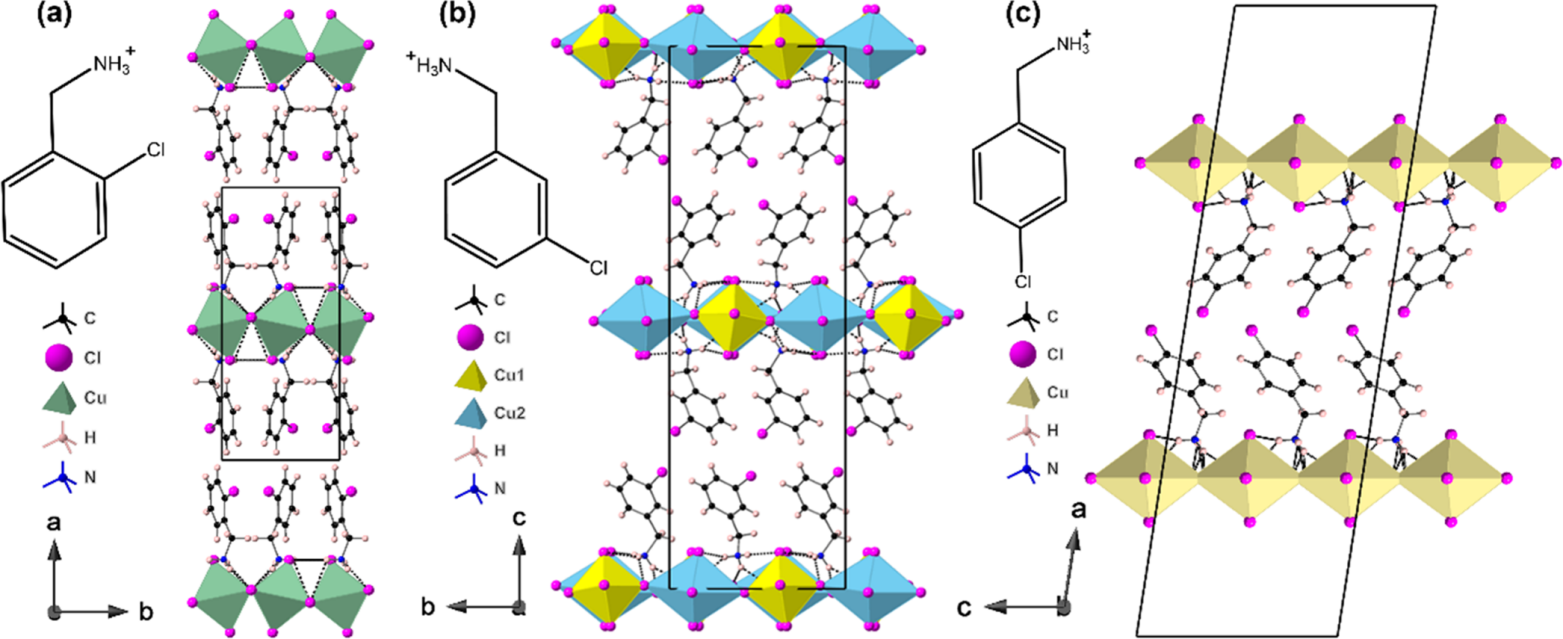
Crystal structures of (a) (2-ClbaH)_2_CuCl_4_, (b) (3-ClbaH)_2_CuCl_4_, and (c) (4-ClbaH)_2_CuCl_4_ at 298 K parallel to the layer direction.

**Figure 2:**
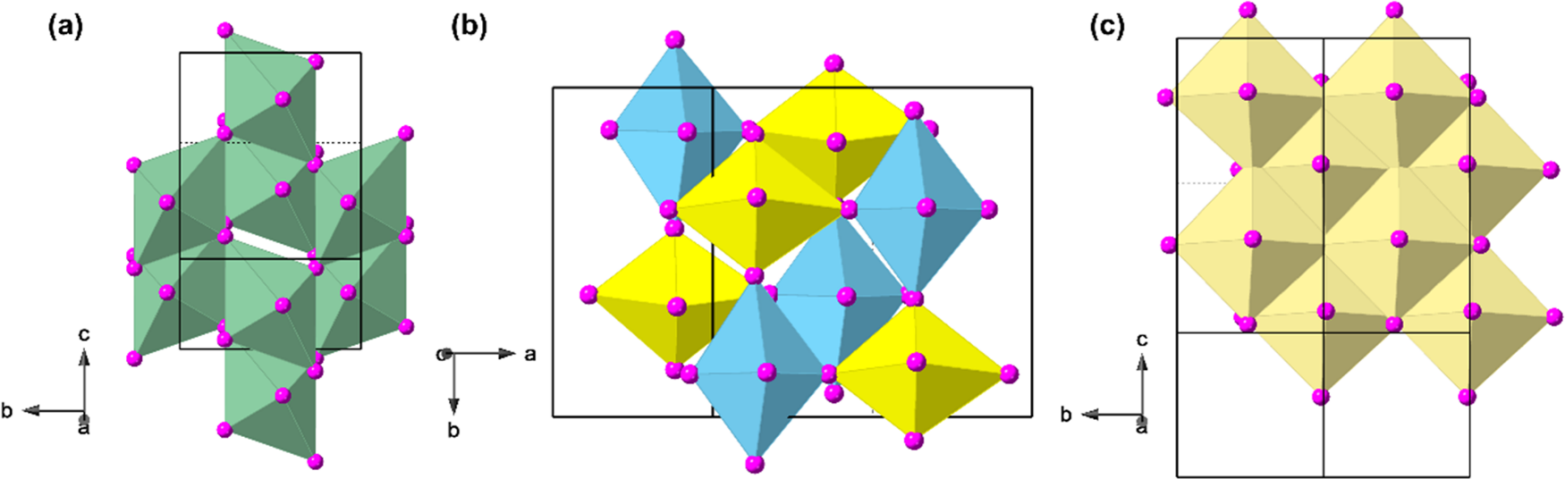
Crystal structures of (a) (2-ClbaH)_2_CuCl_4_, (b) (3-ClbaH)_2_CuCl_4_, and (c) (4-ClbaH)_2_CuCl_4_ at 298 K perpendicular to the layer direction, emphasizing the similarities and differences between the [CuCl_4_]_∞_ layers.

The crystal structures of (2-ClbaH)_2_CuCl_4_, (3-ClbaH)_2_CuCl_4_, and (4-ClbaH)_2_CuCl_4_ are shown in Figure 1. All three structures exhibit the same generic 2D layered perovskite structure type, with a single [CuCl_4_]_∞_ layer of corner-shared CuCl_6_ octahedra separated by a double layer of the protonated chlorobenzylammonium cations, similar to their parent structure (baH)_2_CuCl_4_ (baH^+^ = benzylammonium) and fluorinated counterparts (2/3/4-FbaH)_2_CuCl_4_ (FbaH^+^ = fluorobenzylammonium).^21,27^ Looking in more detail at the unit cell metrics, it can be determined that (2-ClbaH)_2_CuCl_4_, which crystallizes in the centrosymmetric monoclinic system, **P**2_1_/**c**, has a supercell described as *c* × √2*a* × √2*a* relative to the parent DJ-like structure (i.e., just a single [CuCl_4_]_∞_ layer repeat per cell). In fact, this is an example of the most common structure type among layered hybrid perovskites, having the simple Glazer tilt system *a*^–^*a*^–^*c*. At each temperature studied, both (3-ClbaH)_2_CuCl_4_ and (4-ClbaH)_2_CuCl_4_ crystallize in more complex supercells, having two [CuCl_4_]_∞_ layers per cell. Despite having the same polar monoclinic *Cc* space group with a doubled unit cell along the layer direction, the apparent similarity is coincidental, with (3-ClbaH)_2_CuCl_4_ displaying unit cell metrics 2*a* × 2*a* × *c*, relative to the RP-like parent, and (4-ClbaH)_2_CuCl_4_ adopting unit cell metrics *c* × *a* × 2*a*, relative to the RP-like parent. (3-ClbaH)_2_CuCl_4_ displays a complex octahedral tilt system not easily described in the Glazer scheme, whereas (4-ClbaH)_2_CuCl_4_ has no octahedral tilt modes. Both of these structure types are rare, but analogues have been seen in the lead halide family (see Table 3 of ref (7)). A fuller and more systematic discussion of these types of structural features can be found in our recent review.^7^ In the (4-ClbaH)_2_CuCl_4_ structure, the [CuCl_4_]_∞_ sheets display an additional J–T disorder, similar to the known chiral ferromagnets (*R*-MPEA)_2_CuCl_4_ and (*S*-MPEA)_2_CuCl_4_.^28^ In contrast, (3-ClbaH)_2_CuCl_4_ displays a well-behaved structure with two different Cu sites. Figure 2 shows that neighboring [CuCl_4_]_∞_ layers in all three structures are staggered relative to each other in an RP style, with a greater degree of layer shift in the (2-ClbaH)_2_CuCl_4_ structure, described by the larger β angle.

The Cu^2+^ ions in halide perovskite structures exhibit a substantial J–T distortion, resulting in significant variations in the Cu–Cl bond lengths and therefore structural distortions of the CuCl_6_ octahedra, with the shorter in-plane bond *R*_S_, the longer one *R*_L_, and the out-of-plane bond *R*_Z_ (Table 2). To learn more details of the distortions, the degree of octahedral distortion is measured quantitatively using equation .^29^ Among the three compounds, (2-ClbaH)_2_CuCl_4_ displays the largest octahedral and interoctahedral distortions (Cu–Cl–Cu angles).

**Figure 3:**
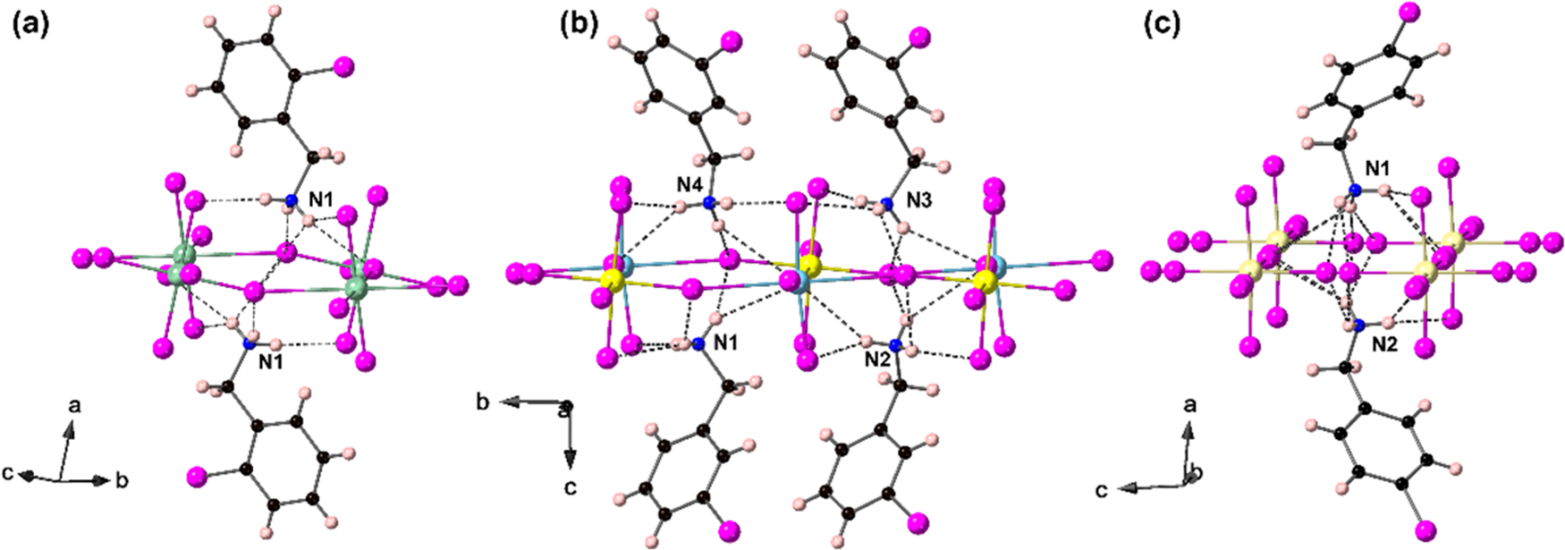
Hydrogen-bonding interactions for (a) (2-ClbaH)_2_CuCl_4_, (b) (3-ClbaH)_2_CuCl_4_, and (c) (4-ClbaH)_2_CuCl_4_ at 298 K.

In all three structures, as shown in Figure 3, the protonated chlorobenzylammonium moieties are ordered and form hydrogen bonds (N–H···Cl) with the inorganic layers [CuCl_4_]_∞_. In the centrosymmetric structure (2-ClbaH)_2_CuCl_4_, the two organic moieties are correlated by an inversion center with stronger hydrogen bonds (N–H···Cl < 2.89 Å), which can be seen as a single crystallographically distinct organic molecule. In the two polar structures, there are four and two distinct organic moieties for (3-ClbaH)_2_CuCl_4_ and (4-ClbaH)_2_CuCl_4_, respectively.

### Second Harmonic Generation Effect

**Figure 4:**
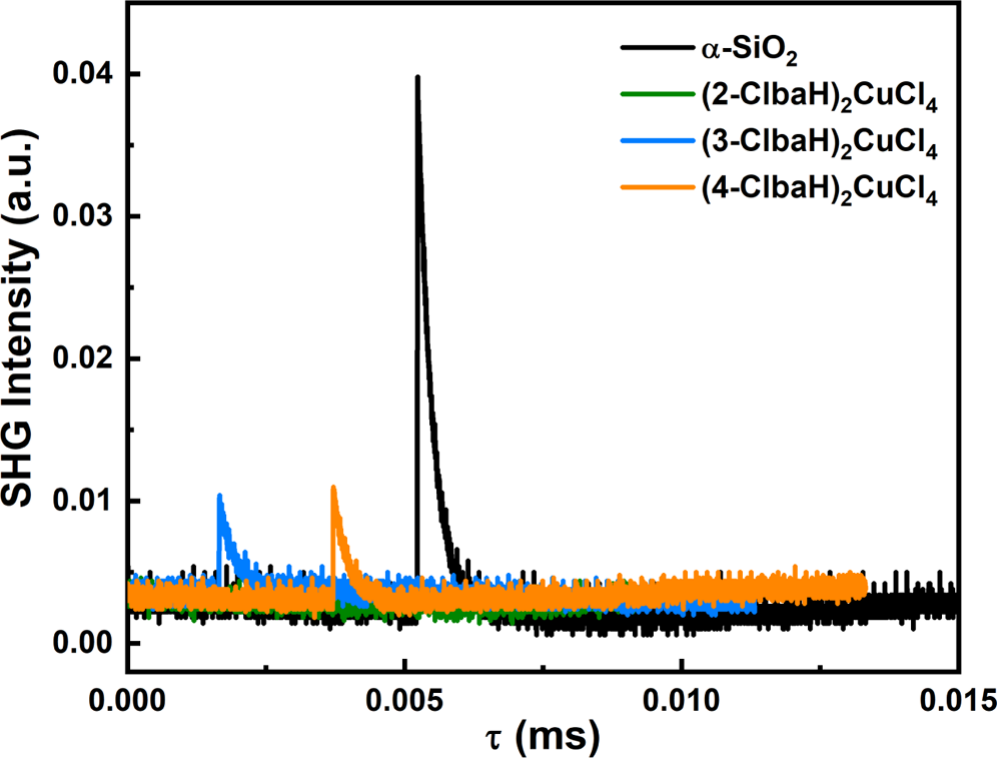
SHG signals of (2-Clba)_2_CuCl_4_, (3-Clba)_2_CuCl_4_, and (4-Clba)_2_CuCl_4_.

To verify the presence or absence of inversion symmetry in the *ortho*-, *meta*-, and *para*-Cl-substituted benzylammonium crystal structures, we conducted second harmonic generation (SHG) experiments. Since the SHG response can only exist in noncentrosymmetric crystal structures, this is a sensitive method to probe the inversion symmetry in the crystal structure. As depicted in Figure 4, (2-Clba)_2_CuCl_4_ is SHG-inactive, while both (3-Clba)_2_CuCl_4_ and (4-Clba)_2_CuCl_4_ show clear SHG signals at room temperature, consistent with the centrosymmetric **P**2_1_/**c** and noncentrosymmetric *Cc* space groups, respectively.

### Dielectric Properties

**Figure 5:**
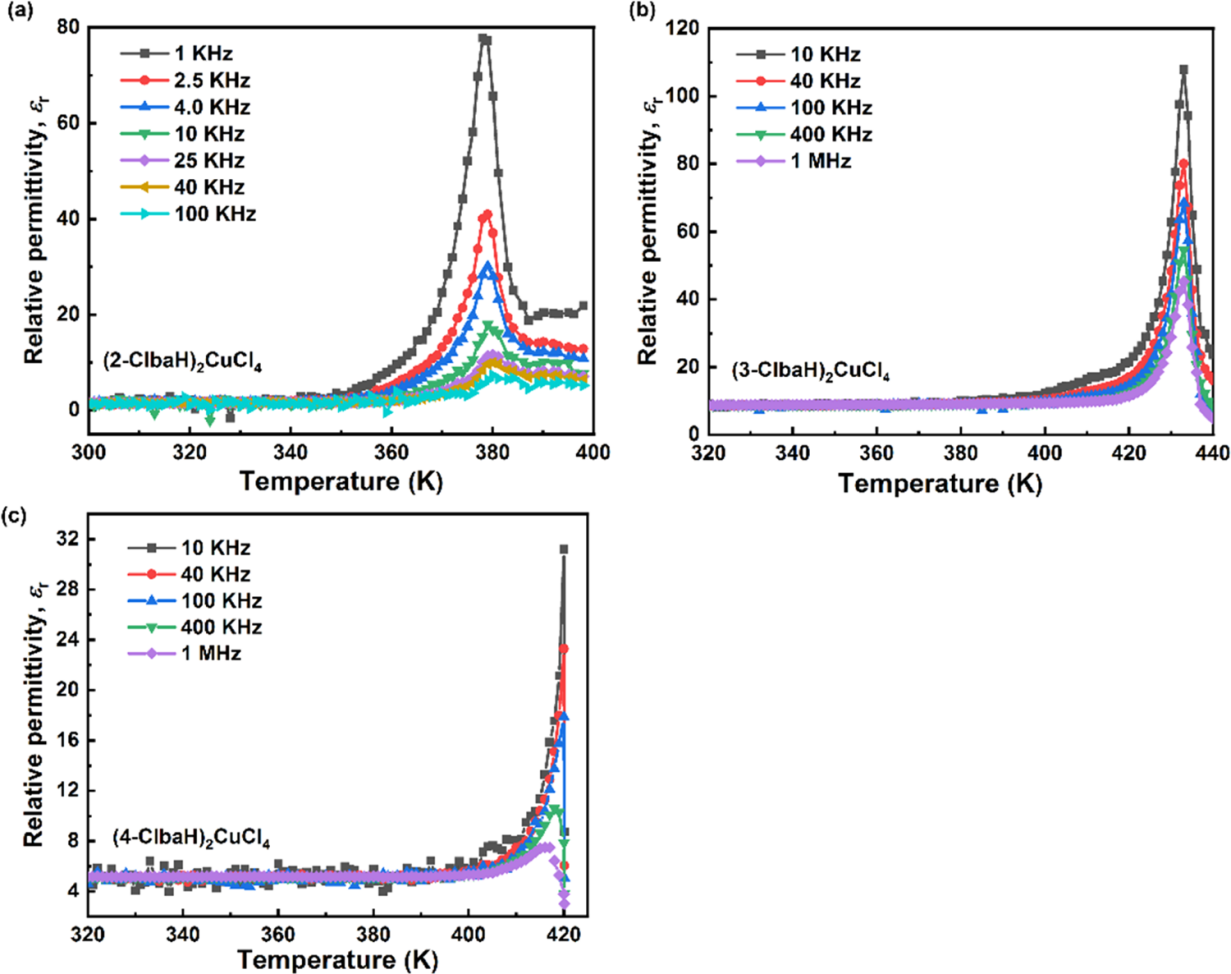
Relative permittivity, ε_r_, as a function of temperature at different frequencies in heating runs of (a) (2-ClbaH)_2_CuCl_4_, (b) (3-ClbaH)_2_CuCl_4_, and (c) (4-ClbaH)_2_CuCl_4_.

Powder-pressed pellets were measured at various frequencies to investigate the dielectric properties of all three compounds. The dielectric permittivity usually displays large anomalies in the vicinity of the phase transition temperature.^30^ As shown in Figure 5, upon heating, the relative permittivity (ε_r_) data show anomalies at 379, 433, and 420 K for (2-ClbaH)_2_CuCl_4_, (3-ClbaH)_2_CuCl_4_, and (4-ClbaH)_2_CuCl_4_, respectively, suggesting the existence of phase transitions in the three compounds. The peak temperatures of the dielectric peaks show no apparent shift under various frequencies, suggesting that this dispersion is not due to dielectric relaxation. In the case of (2-ClbaH)_2_CuCl_4_, it is likely that the phase transition involves a change of the octahedral tilt system to a higher-symmetry centrosymmetric polymorph. This phenomenon has been observed in hybrid lead systems, for example, [4-fluorobenzylammonium]_2_PbCl_4_ displays a centrosymmetric-to-centrosymmetric phase transition with significant dielectric anomalies.^31^ However, the sharp dielectric peaks of the two polar compounds support the occurrence of polar-to-nonpolar phase transitions, which may be induced by the ordered–disordered dynamic motions of organic moieties in hybrid perovskites.^31,32^ The variation of ε_r_ on cooling for the two polar compounds also shows prominent peaks under different frequencies, strongly supporting their reversible polar-to-nonpolar phase transitions (Figure S4). To the best of our knowledge, such a high polar-to-nonpolar phase transition temperature 433 K of (3-ClbaH)_2_CuCl_4_ is the highest in 2D copper(II) perovskites and significantly larger than [3,3-difluorocyclobutylammonium]_2_CuCl_4_ (380 K),^22^ (C_6_H_5_CH_2_CH_2_NH_3_)_2_CuCl_4_ (340 K),^33^ and [C_6_H_5_(CH_2_)_4_NH_3_]_2_CuCl_4_ (143 K).^34^

### Magnetic Properties

**Figure 6:**
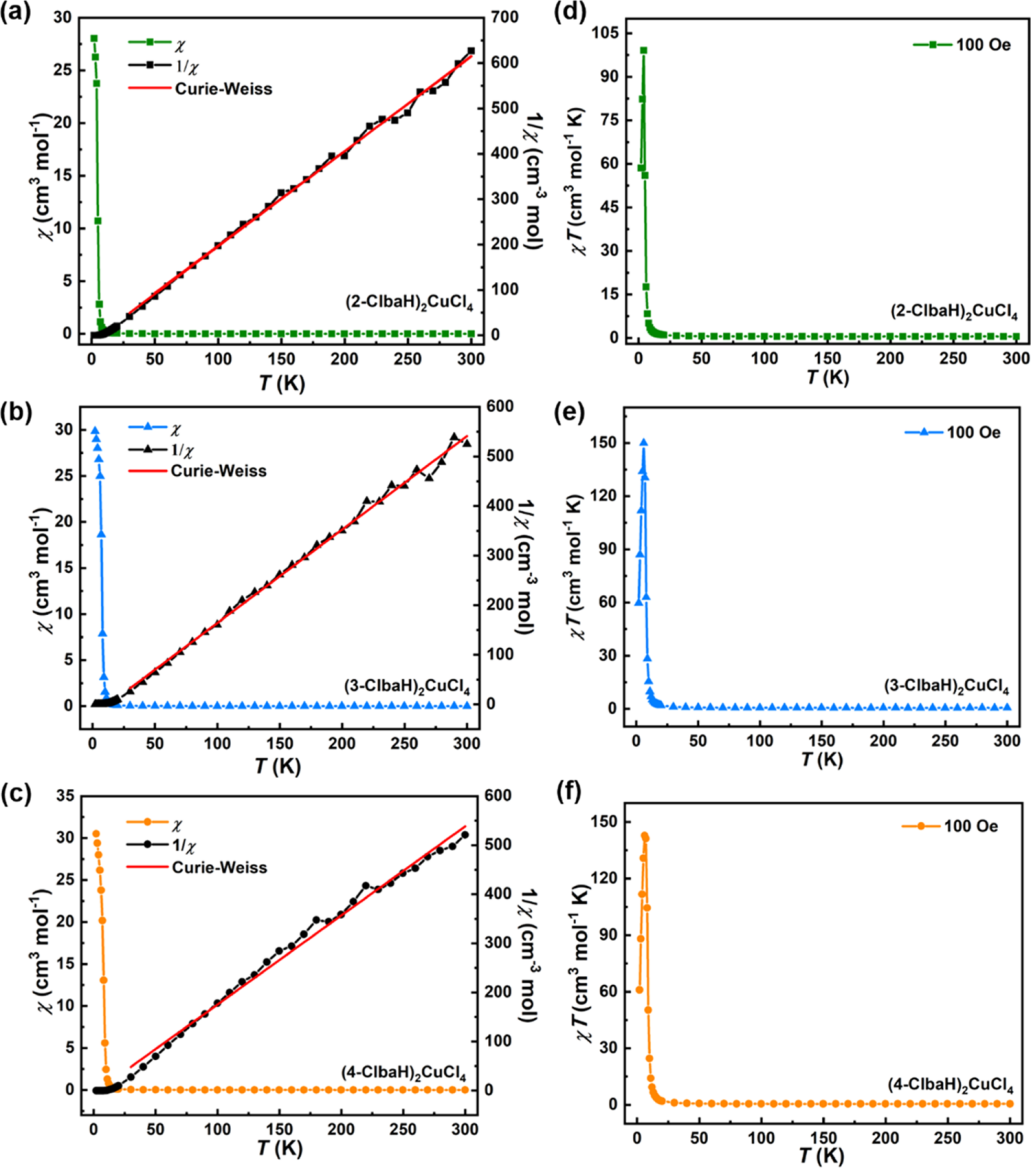
Magnetic susceptibility (χ) and its inverse 1/χ with the Curie–Weiss fit (red line) in the region 30–300 K for (a) (2-ClbaH)_2_CuCl_4_, (b) (3-ClbaH)_2_CuCl_4_, and (c) (4-ClbaH)_2_CuCl_4_. χ*T* versus *T* for (d) (2-ClbaH)_2_CuCl_4_, (e) (3-ClbaH)_2_CuCl_4_, and (f) (4-ClbaH)_2_CuCl_4_, respectively.

**Figure 7:**
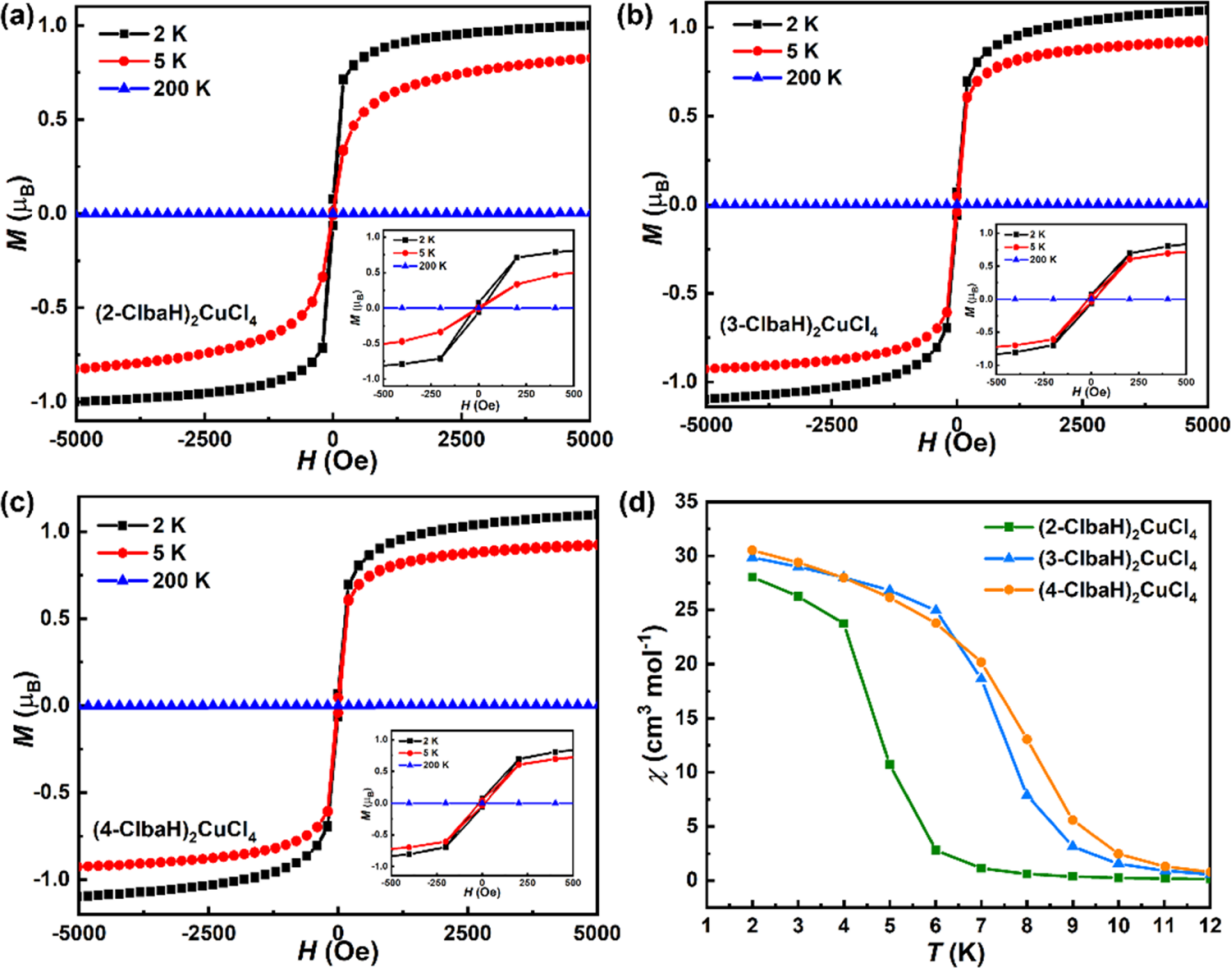
Ferromagnetic hysteresis loops of (a) (2-ClbaH)_2_CuCl_4_, (b) (3-ClbaH)_2_CuCl_4_, and (c) (4-ClbaH)_2_CuCl_4_ at 2, 5, and 200 K. (d) The susceptibility χ in the vicinity of the transition.

SQUID magnetometry was used to explore the magnetic properties of the three compounds. As shown in Figure 6 a–c, the magnetic susceptibility (χ) is displayed as a function of temperature *T* during field cooling. χ shows a gradual increase in cooling from 300 K to around 10 K, below which it increases very steeply, with a curvature that suggests a tendency toward saturation as the temperature is further reduced. This leads to a peak in the product χ*T* shown in Figure 6d–f, which otherwise has a very slight upward trend over much of the range as the temperature falls. These observations are all strongly indicative of a system dominated by ferromagnetic interactions with a transition to an ordered ferromagnetic state at low temperature, with no indication of moment compensation due to antiferromagnetic correlations at any temperature. This is further supported by the magnetization *M* vs *T* plots taken at low temperature shown in Figure 7a–c. At the lowest temperatures measured, these display very soft ferromagnetic behavior, with near-saturation values at the maximum field measured of approximately 1.00, 1.02, and 1.03 μ_B_ for (2-ClbaH)_2_CuCl_4_, (3-ClbaH)_2_CuCl_4_, and (4-ClbaH)_2_CuCl_4_, respectively. This is comparable to the expected pure spin value (1 μ_B_) for an *S* = 1/2 system using *g* = 2.^35,36^ This recovery of the full possible moment confirms the onset of ferromagnetic order at low temperature. The insets of Figure 7 indicate a small amount of hysteresis present in the curves. Although we have not attempted to quantify the coercivity, it is clear that these are very soft magnetically.

The inverse susceptibility (1/χ) plots (Figure 6) have an approximately linear dependence on high temperature and have been fitted to the Curie–Weiss law in the range of 30–300 K. The extracted Curie constants (*C*) for (2-ClbaH)_2_CuCl_4_, (3-ClbaH)_2_CuCl_4_, and (4-ClbaH)_2_CuCl_4_, are 0.50, 0.53, and 0.54 cm^3^ mol^–1^ K, respectively, and the corresponding Weiss constants (θ) are 6.8, 12.9, and 3.3 K, the latter again being consistent with ferromagnetic correlations. The derived effective moments (μ_eff_) are 2.004, 2.059, and 2.078 μ_B_, which, by making use of the moment per Cu site *M* taken from the saturation curves, leads to ratios μ_eff_/*M* of 2.004, 2.02, and 2.02. In an idealized measurement for a pure *S* = 1/2 spin system, this ratio should be , under the assumption of a temperature-independent Curie constant. The interpretation of experimental values of μ_eff_ should, however, always be treated with some caution^37^ and may overlook potential temperature-dependent contributions, including subtle modifications to the ligand field, spin–orbit interactions, and exchange anisotropy. The moment obtained from the low-temperature saturated ordered ferromagnetic state is a more reliable measure of the ground state, which indeed agrees very well with that expected for the *S* = 1/2 system. The naive application of the Curie–Weiss law leads to values of the Landé factors (*g*) of 2.314, 2.379, and 2.400 for (2-ClbaH)_2_CuCl_4_, (3-ClbaH)_2_CuCl_4_, and (4-ClbaH)_2_CuCl_4_, respectively, which although are within the predicted range for one Cu^2+^ ion with *S* = 1/2,^35,36,38^ likely to be an overestimate given the slightly inflated values of μ_eff_.

To compare the influence of the structure on the ferromagnetic ordering temperature *T*_c_, we plot in Figure 7d the susceptibility χ in the vicinity of transition. (4-ClbaH)_2_CuCl_4_ shows the onset of order at the highest temperature around 10 K, with (3-ClbaH)_2_CuCl_4_ ordering at a slightly lower temperature. By contrast, (2-ClbaH)_2_CuCl_4_ orders at a significantly lower temperature around 6 K. Clearly, the Curie temperature is determined mainly by the inorganic framework [CuCl_4_]_∞_: of the three compounds, (2-ClbaH)_2_CuCl_4_ possesses the largest octahedral and interoctahedral distortions (Table 2) and has the lowest *T*_c_. This may be the most significant factor, but differences in the Jahn–Teller orbital ordering may also have an effect.

The measurements clearly demonstrate that all three compounds possess dominant ferromagnetic interactions within the [CuCl_4_]_∞_ layers, which are similar to previously reported 2D layered copper(II) perovskites.^35,36,38^ The relationship between the ferromagnetic order and the structure in inorganic–organic hybrid perovskites containing [CuCl_4_]_∞_ layers has recently been explored using DFT calculations.^39^ Although the organic spacer layers considered are different from those studied here, the systems share the common motif of [CuCl_4_]_∞_ layers, with J–T distortions leading to alternating in-plane bond lengths at nearest-neighbor Cu sites. These calculations considered both the full structures and also a single [CuCl_4_]_∞_ layer sandwiched between the organic cations. Perhaps not surprisingly, it was found that exchange constants that were only slightly different in the single-layer systems compared to those in the bulk and all materials (3D and 2D) were found to display strong in-plane ferromagnetic correlations arising from the near-180° superexchange pathway coupled with the alternating nearest-neighbor bond length. The out-of-plane exchange coupling was very small and generally antiferromagnetic in nature. A moment of around 1 μ_B_ per Cu was predicted, comparable to that found in our data, though interestingly, the apparent strong covalency suggested that around a third of this could be associated with the Cl sites.

It is interesting to consider the driver for ferromagnetic order in these materials since for an isotropic Heisenberg model the occurrence of long-range order is forbidden by the Merin–Wagner theorem in 2D rotationally invariant systems.^40^ The breaking of this symmetry is unlikely to come from the exchange anisotropy due to the relatively small out-of-plane coupling. In the DFT calculations, the inclusion of spin–orbit coupling suggested the existence of a single ion anisotropy capable of precipitating 2D ferromagnetic order,^39^ giving rise to an additional large unquenched moment of 0.1 μ_B_ at the Cu site. Calculations further suggested that an in-plane orientation of the moment would be preferred. To explore further, the moment orientation using magnetometry will require the growth of single crystals of the materials, but probing the anisotropy in these materials is likely to significantly enhance our understanding of the origins of magnetic order. The importance of these materials as tunable 2D ferromagnets can be viewed in the context of the significant interest in the monolayer limit of materials such as CrI_3_^41^ and Cr_2_Ge_2_Te_6_^42^ and the present highly 2D systems might be expected to have a good potential for cleavage if suitable single crystals could be grown.

### Optical Properties

**Figure 8:**
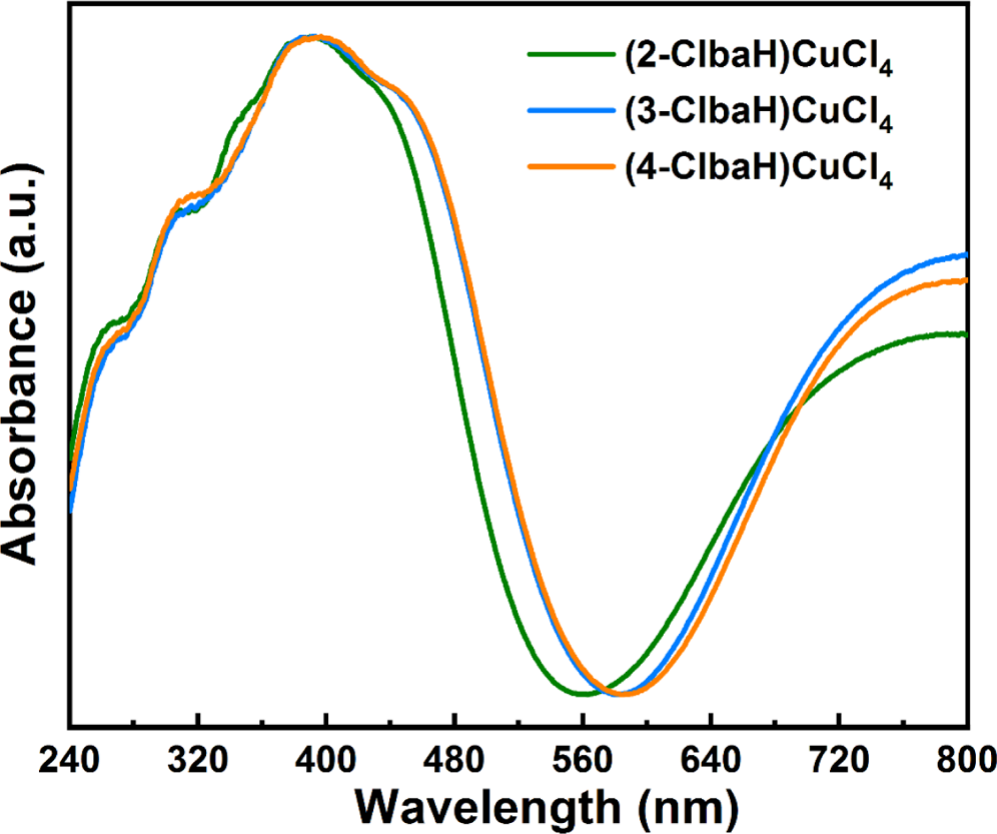
UV–vis absorption spectra of (2-ClbaH)_2_CuCl_4_, (3-ClbaH)_2_CuCl_4_, and (4-ClbaH)_2_CuCl_4_.

The optical properties of the three materials were explored by ultraviolet–visible (UV–vis) absorption spectra in the solid state. In the UV–vis absorption spectra at ambient temperature (Figure 8), they display similar absorption behavior with a sharp absorption edge at about 540, 570, and 580 nm for (2-ClbaH)_2_CuCl_4_, (3-ClbaH)_2_CuCl_4_, and (4-ClbaH)_2_CuCl_4_, respectively. The strongest absorption peaks in these spectra at around 390 nm can be attributed to the excitation of an electron from the valence to the conduction band, as previously studied for [CuCl_4_]_∞_ perovskites,^11^ suggesting a direct band-gap characteristic.

**Figure 9:**
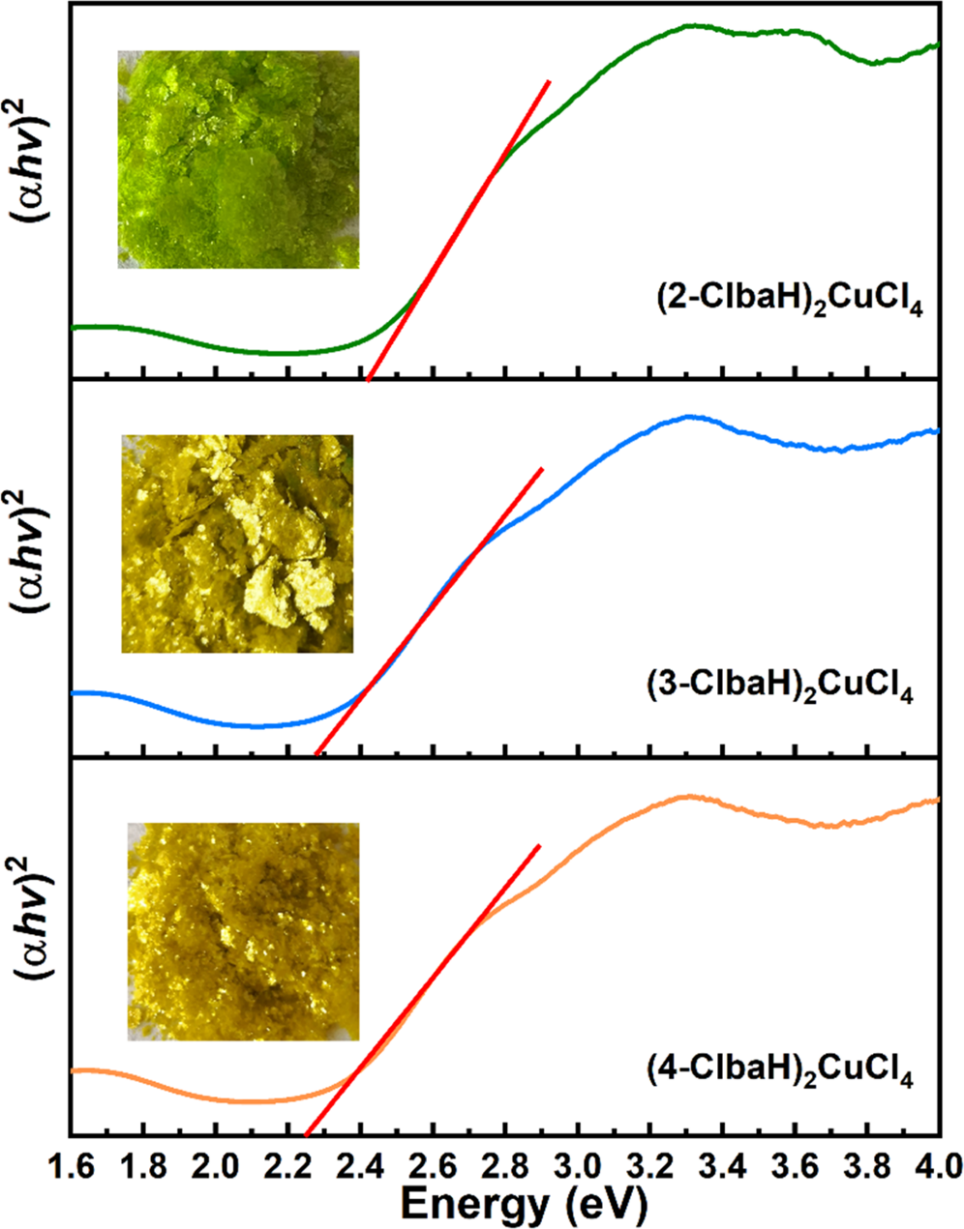
Tauc plots and the corresponding crystals of (2-ClbaH)_2_CuCl_4_, (3-ClbaH)_2_CuCl_4_, and (4-ClbaH)_2_CuCl_4_.

Although the band-gap values determined from Tauc plots have a significant error, we can roughly predict their optical band gaps.^43^ The band gaps obtained from Tauc plots (Figure 9) are about 2.43 eV for (2-ClbaH)_2_CuCl_4_, 2.28 eV for (3-ClbaH)_2_CuCl_4_, and 2.25 eV for (4-ClbaH)_2_CuCl_4_, accompanied by a small regulative magnitude of 0.18 eV, which are comparable to that observed in other perovskites, e.g., 2.48 eV for (CH_3_NH_3_)_2_CuCl_4_^11^ and 2.16 eV for (BED)_2_CuCl_6._^16^ The interoctahedral distortions of the inorganic framework play an important role in determining the band gap of layered hybrid halide perovskites.^44^ The two polar compounds (3-ClbaH)_2_CuCl and (4-ClbaH)_2_CuCl_4_ possess narrower band gaps, in agreement with their smaller interoctahedral distortions (larger Cu–Cl–Cu angles; Table 2). As shown in Figure 9, the colors of the three compounds change from bright green ((2-ClbaH)_2_CuCl_4_) to green-yellow ((3-ClbaH)_2_CuCl_4_) and then to yellow ((4-ClbaH)_2_CuCl_4_), perfectly corresponding to their band gaps.

## Conclusions

In summary, we have explored the use of chlorine-substituted benzylamine isomers in templating differing structural features in layered copper chloride perovskites. While the (2-ClbaH)_2_CuCl_4_ isomer displays a common, centrosymmetric variant of this generic structure type, both the 3- and 4-substituted analogues display more complex and distinct noncentrosymmetric structures. SHG measurements confirm the noncentrosymmetric nature of the latter isomers, while dielectric and magnetic measurements confirm unusually high-temperature transitions to assume higher-symmetry phases and ferromagnetism at low temperatures, respectively. The trends in magnetic behavior and optical properties can be rationalized based on trends in the distortions of the [CuCl_4_]_∞_ layers. The introduction of chlorine atoms in the benzylamine structure is therefore shown to be a useful strategy to modify crystal structures and physical properties in hybrid copper perovskites. This research further highlights 2D hybrid copper halide perovskites as an excellent platform for the development of innovative multifunctional materials.

## Supporting Information Available

Rietveld refinements of PXRD data, crystallographic data at 173 K, thermogravimetric analysis data, relative permittivity data in cooling runs, and hydrogen-bonding details (PDF)
Crystallographic data (CIF)
Crystallographic data (CIF)

## Supplementary Material

cm2c00107_si_001.pdf

cm2c00107_si_002.cif

cm2c00107_si_003.cif

## Notes

The research data supporting this publication can be accessed at https://doi.org/10.17630/50f4f521-80ec-4027-8676-d82c46ccc7ca.
