# Supplementary material for: Polarity and Ferromagnetism in Two-Dimensional Hybrid Copper Perovskites with Chlorinated Aromatic Spacers
Source: Chem Mater. 2022 Feb 21;34(5):2458–67. doi: 10.1021/acs.chemmater.2c00107 (PMC9008537; doi:10.1021/acs.chemmater.2c00107)
Supplement: Supplementary file 1 — cm2c00107_si_001.pdf [file cm2c00107_si_001.pdf]

Polarity and ferromagnetism in two-dimensional hybrid copper perovskites with chlorinated aromatic spacers

Ceng Han,[†] Alasdair J. Bradford,[†,‡] Jason A. McNulty,[†] Weiguo Zhang,[§] P. Shiv Halasyamani,[§] Alexandra M. Z. Slawin,[†] Finlay D. Morrison,[†] Stephen L. Lee,[‡] and Philip Lightfoot*,[†]

[†]School of Chemistry and EaStChem, University of St Andrews, St Andrews, KY16 9ST, United Kingdom

[‡]School of Physics, University of St Andrews, St Andrews, Fife, KY16 9SS, United Kingdom

[§]Department of Chemistry, University of Houston, Houston, Texas 77204, United States

*e-mail: pl@st-andrews.ac.uk

Supplementary Information

Figure S1. The full-range (left) and expanded (right) PXRD data for (a) (2-ClbaH)$_2$CuCl$_4$, (b) (3-ClbaH)$_2$CuCl$_4$ and (c) (4-ClbaH)$_2$CuCl$_4$.

Figure S2. Full-range and expanded (inset) Rietveld plot (PXRD) for (a) (2-ClbaH)$_2$CuCl$_4$, (b) (3-ClbaH)$_2$CuCl$_4$ and (c) (4-ClbaH)$_2$CuCl$_4$. Note that there is a significant preferred orientation. In addition, only unit cell and profile parameters were refined, not atomic parameters. The aim here is merely to demonstrate phase purity, which is confirmed by the elemental analysis.

Figure S3. Thermogravimetric analysis (TGA) data for (a) (2-ClbaH)$_2$CuCl$_4$, (b) (3-ClbaH)$_2$CuCl$_4$ and (c) (4-ClbaH)$_2$CuCl$_4$.

Figure S4. Relative permittivity, ε_r, as a function of temperature at 10 KHz,100 KHz and 1 MHz in cooling runs of (a) (3-Clba)$_2$CuCl$_4$ and (b) (4-Clba)$_2$CuCl$_4$.

Figure S5. Thermal evolution of the lattice metrics obtained from Rietveld refinement for (a) (3-ClbaH)$_2$CuCl$_4$ at the range 290 K to 440 K and (b) (4-ClbaH)$_2$CuCl$_4$ at the range 92 K to 440 K.

Table S1. Crystal and Refinement Data for $(2\text{-ClbaH})_2CuCl_4$, $(3\text{-ClbaH})_2CuCl_4$ and $(4\text{-ClbaH})_2CuCl_4$ at 173 K.

Table S2 Hydrogen bond lengths (Å) and angles (°) for $(2\text{-ClbaH})_2CuCl_4$ at 173 K.

Table S3. Hydrogen bond lengths (Å) and angles (°) for $(2\text{-ClbaH})_2CuCl_4$ at 298 K.

Table S4. Hydrogen bond lengths (Å) and angles (°) for $(3\text{-ClbaH})_2CuCl_4$ at 173 K.

Table S5. Hydrogen bond lengths (Å) and angles (°) for $(3\text{-ClbaH})_2CuCl_4$ at 298 K.

Table S6. Hydrogen bond lengths (Å) and angles (°) for $(4\text{-ClbaH})_2CuCl_4$ at 173 K.

Table S7. Hydrogen bond lengths (Å) and angles (°) for $(4\text{-ClbaH})_2CuCl_4$ at 298 K.

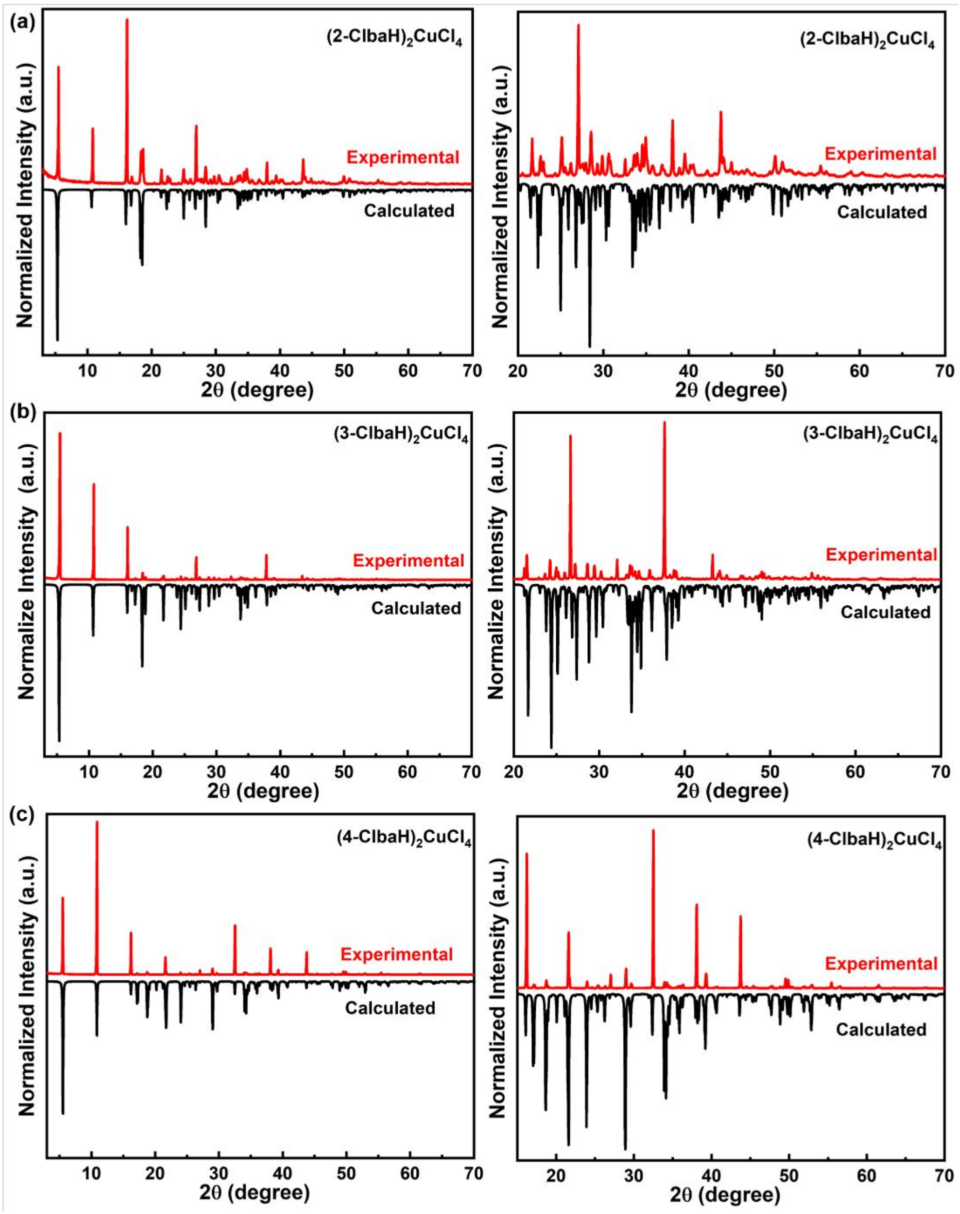

Figure S1. The full-range (left) and expanded (right) PXRD data for (a) (2-

ClbaH)$_2$CuCl$_4$, (b) (3-ClbaH)$_2$CuCl$_4$ and (c) (4-ClbaH)$_2$CuCl$_4$.

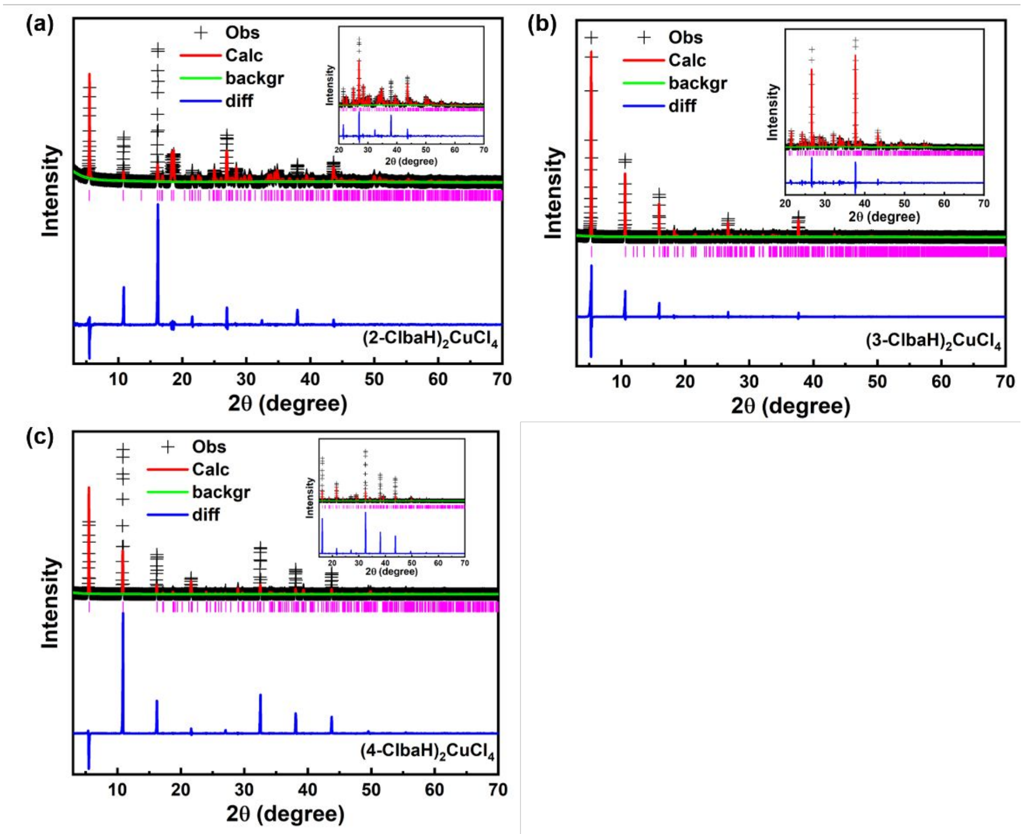

Figure S2. Full-range and expanded (inset) Rietveld plot (PXRD) for (a) (2-ClbaH)$_2$CuCl$_4$, (b) (3-ClbaH)$_2$CuCl$_4$ and (c) (4-ClbaH)$_2$CuCl$_4$. Note that there is a significant preferred orientation. In addition, only unit cell and profile parameters were refined, not atomic parameters. The aim here is merely to demonstrate phase purity, which is confirmed by the elemental analysis.

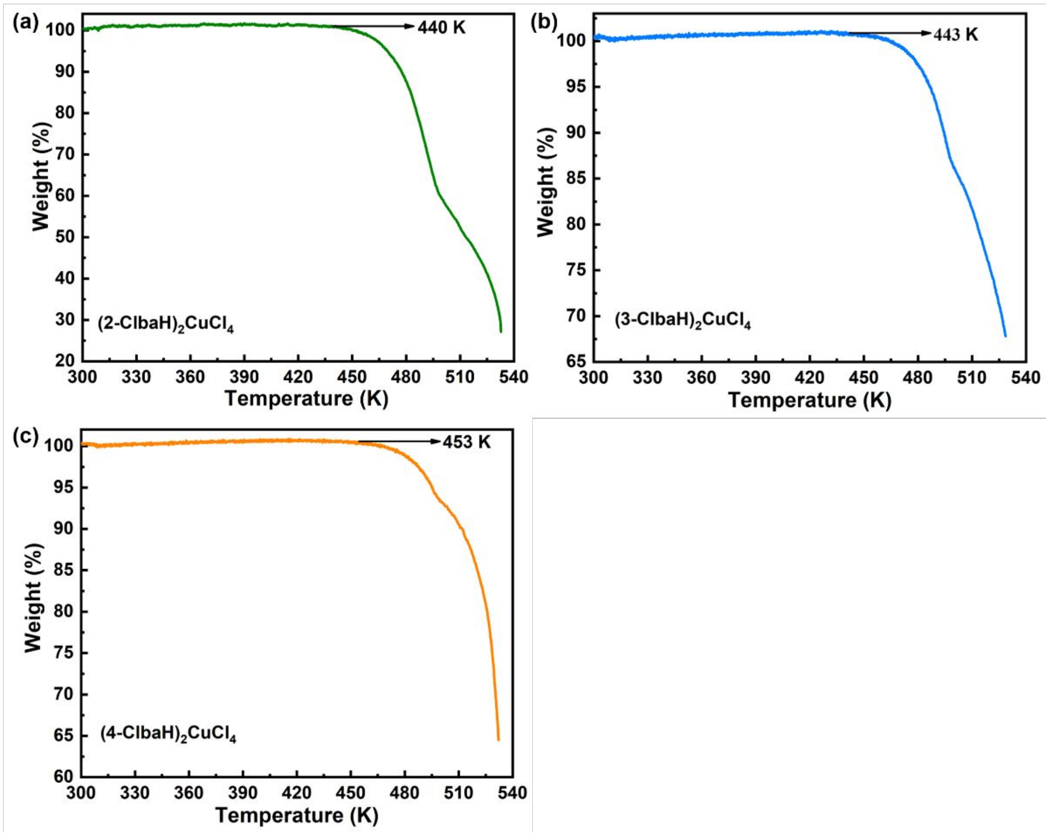

Figure S3. Thermogravimetric analysis (TGA) data for (a) (2-ClbaH)$_2$CuCl$_4$, (b) (3-ClbaH)$_2$CuCl$_4$ and (c) (4-ClbaH)$_2$CuCl$_4$.

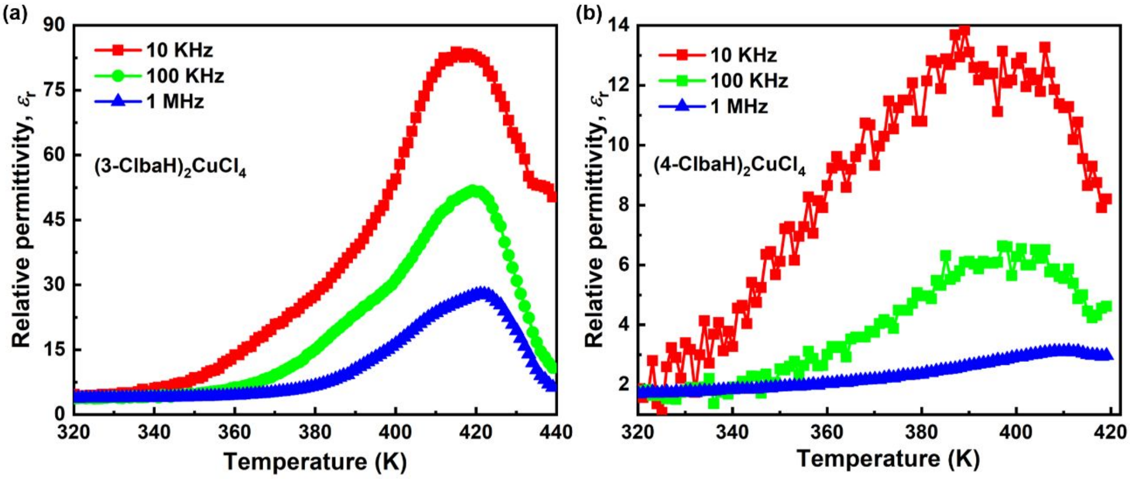

Figure S4. Relative permittivity, ε_r, as a function of temperature at 10 KHz,100 KHz and 1 MHz in cooling runs of (a) (3-Clba)$_2$CuCl$_4$ and (b) (4-Clba)$_2$CuCl$_4$.

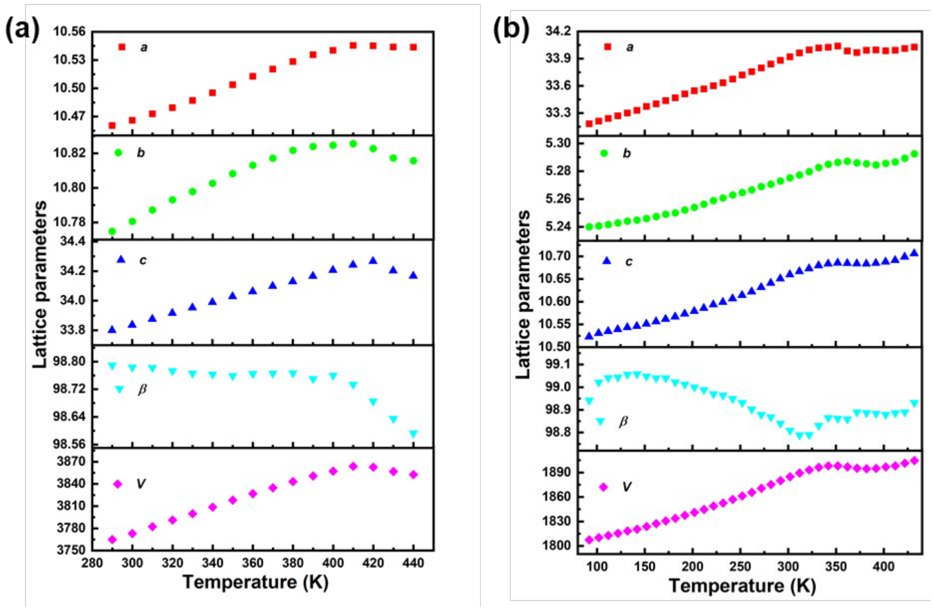

Figure S5. Thermal evolution of the lattice metrics obtained from Rietveld refinement for (a) $(3\text{-ClbaH})_2CuCl_4$ within the range 290 K to 440 K and (b) $(4\text{-ClbaH})_2CuCl_4$ withing the range 92 K to 440 K.

The single-crystal XRD of $(3\text{-ClbaH})_2CuCl_4$ was measured above 400 K. However, the structure at high temperature has not been determined, because the diffraction in the high temperature phase is too weak. In order to get more details of the phase transitions, high-resolution synchrotron X-ray powder diffraction (SXRD) data for $(3\text{-ClbaH})_2CuCl_4$ from 290 K to 440 K and $(4\text{-ClbaH})_2CuCl_4$ from 92 K to 440 K were collected at Beamline I11 Diamond Light Source, UK. Thermal evolutions of unit cell parameters were derived from GSAS refinement of the SXRD data (Figure S5). We can conclude that unit cell parameters a, b, c and volume of $(3\text{-ClbaH})_2CuCl_4$ and $(4\text{-ClbaH})_2CuCl_4$ increase when heated to 410 and 350 K, respectively, and subsequently show decreasing trends with further heating, indicating that the powder samples are likely decomposing on heating. The β angles in both structures display unusual trends, possibly due to the complexity of the structures. We cannot get any further details of phase transitions from the powder data because of the intrinsic complexity of the structures and the decomposition of samples during the measurement.

Table S1. Crystal and Refinement Data for (2-ClbaH)$_2$CuCl$_4$, (3-ClbaH)$_2$CuCl$_4$ and (4-ClbaH)$_2$CuCl$_4$ at 173 K.

compound	(2-ClbaH)$_2$CuCl$_4$	(3-ClbaH)$_2$CuCl$_4$	(4-ClbaH)$_2$CuCl$_4$
**formula**	$C_{14}H_{18}N_2CuCl_6$	$C_{14}H_{18}N_2CuCl_6$	$C_{14}H_{18}N_2CuCl_6$
**formula weight**	490.54	490.54	490.54
**colour/habit**	Green/Platelet	Yellow/Platelet	Yellow/Platelet
**crystal size (mm^3)**	0.27 × 0.18 × 0.05	0.29 × 0.24 × 0.07	0.50 × 0.50 × 0.02
**crystal system**	Monoclinic	Monoclinic	Monoclinic
**space group**	$P2_1/c$	*Cc*	*Cc*
a **(Å)**	16.9185(11)	10.4123(7)	33.404(2)
b **(Å)**	7.0547(5)	10.7426(7)	5.2452(4)
c **(Å)**	8.0522(5)	33.613(2)	10.5586(8)
α **(deg)**	90	90	90
β **(deg)**	102.164(12)	98.904(4)	99.130(4)
γ **(deg)**	90	90	90
V **(Å^3)**	939.49(12)	3712.5(4)	1826.5(2)
Z	2	8	4
ρ_{calc} **(mg/m^3)**	1.734	1.755	1.784
μ **(mm^{-1})**	2.014	2.038	2.072
**F(000)**	494	1976	988
**No. of reflns collected**	7323	18000	8974
**independent reflns**	2204 [*R*(int) = 0.124]	8276 [*R*(int) = 0.0428]	4198 [*R*(int) = 0.0357]
**goodness of fit**	1.094	1.092	0.887
**final *R* indices ($I > 2\sigma(I)$)**	R_1 = 0.1210 wR_2 = 0.3061	R_1 = 0.0354 wR_2 = 0.0891	R_1 = 0.0295 wR_2 = 0.0563
**largest diff. peak/hole (e Å^{-3})**	2.345/-1.214	1.029/-0.544	0.282/-0.357

Table S2. Hydrogen bond lengths (Å) and angles (°) for $(2\text{-ClbaH})_2CuCl_4$ at 173 K.

D-H…A	d(D-H)	d(H…A)	d(D…A)	∠(DHA)
N(1)-H(1A)...Cl(1)#2	0.89	2.46	3.277(11)	153.2
N(1)-H(1A)...Cl(2)#2	0.89	2.88	3.337(13)	113.6
N(1)-H(1B)...Cl(2)#3	0.89	2.35	3.235(13)	169.9
N(1)-H(1C)...Cl(2)	0.89	2.53	3.235(13)	136.8

Symmetry transformations used to generate equivalent atoms:

#1 -x+1,-y+2,-z+1; #2 -x+1,-y+1,-z+1; #3 x,-y+3/2,z+1/2

Table S3. Hydrogen bond lengths (Å) and angles (°) for $(2\text{-ClbaH})_2CuCl_4$ at 298 K.

D-H…A	d(D-H)	d(H…A)	d(D…A)	∠(DHA)
N(1)-H(1A)...Cl(1)#2	0.89	2.37	3.222(7)	160.0
N(1)-H(1B)...Cl(1)	0.89	2.43	3.218(6)	147.1
N(1)-H(1B)...Cl(1)#3	0.89	2.89	3.308(6)	110.4
N(1)-H(1B)...Cl(2)#1	0.89	2.80	3.419(7)	127.7
N(1)-H(1C)...Cl(2)#3	0.89	2.37	3.260(6)	175.9

Symmetry transformations used to generate equivalent atoms:

#1 -x+1,-y+1,-z+2; #2 x,-y+1/2,z-1/2; #3 -x+1,y+1/2,-z+3/2

Table S4. Hydrogen bond lengths (Å) and angles (°) for $(3\text{-ClbaH})_2CuCl_4$ at 173 K.

D-H…A	d(D-H)	d(H…A)	d(D…A)	∠(DHA)
N(1)-H(1A)...Cl(3)	0.89	2.48	3.321(9)	158.1
N(1)-H(1B)...Cl(1)	0.89	2.78	3.366(9)	124.3
N(1)-H(1B)...Cl(5)#1	0.89	2.61	3.386(9)	146.1
N(1)-H(1C)...Cl(2)#2	0.89	2.68	3.318(9)	129.9
N(1)-H(1C)...Cl(6)	0.89	2.66	3.356(8)	135.7
N(2)-H(2A)...Cl(3)#3	0.89	2.57	3.376(9)	151.7
N(2)-H(2A)...Cl(5)#4	0.89	2.87	3.434(8)	122.7
N(2)-H(2B)...Cl(2)	0.89	2.55	3.354(9)	150.7
N(2)-H(2B)...Cl(8)	0.89	2.92	3.459(8)	120.8
N(2)-H(2C)...Cl(6)	0.89	2.38	3.261(8)	168.6
N(3)-H(3A)...Cl(1)#5	0.89	2.55	3.268(9)	138.6
N(3)-H(3A)...Cl(4)#5	0.89	2.70	3.442(8)	141.3
N(3)-H(3B)...Cl(2)#2	0.89	2.72	3.431(8)	137.4
N(3)-H(3B)...Cl(8)#2	0.89	2.71	3.419(9)	137.9
N(3)-H(3C)...Cl(7)	0.89	2.34	3.212(8)	164.9
C(13)-H(13)...Cl(6)	0.93	2.92	3.764(9)	151.5
C(18)-H(18)...Cl(4)	0.93	2.90	3.739(8)	150.4
N(4)-H(4A)...Cl(4)	0.89	2.41	3.244(8)	157.1
N(4)-H(4B)...Cl(1)#4	0.89	2.67	3.346(8)	133.3
N(4)-H(4B)...Cl(5)#6	0.89	2.74	3.480(8)	141.1

N(4)-H(4C)...Cl(7)#4	0.89	2.75	3.450(8)	136.9
N(4)-H(4C)...Cl(8)#4	0.89	2.53	3.309(8)	146.1

Symmetry transformations used to generate equivalent atoms:
#1 x+1/2,y-1/2,z; #2 x-1/2,y-1/2,z; #3 x-1/2,y+1/2,z; #4 x+1/2,y+1/2,z; #5 x-1,y,z; #6 x+1,y,z

Table S5. Hydrogen bond lengths (Å) and angles (°) for $(3\text{-ClbaH})_2CuCl_4$ at 298 K.

D-H…A	d(D-H)	d(H…A)	d(D…A)	∠(DHA)
N(1)-H(1A)...Cl(6)	0.89	2.36	3.243(17)	170.6
N(1)-H(1B)...Cl(2)#1	0.89	2.79	3.439(17)	130.9
N(1)-H(1B)...Cl(8)#1	0.89	2.70	3.443(18)	142.1
N(1)-H(1C)...Cl(1)#2	0.89	2.60	3.264(19)	131.7
N(1)-H(1C)...Cl(4)#2	0.89	2.64	3.429(17)	147.6
N(2)-H(2A)...Cl(6)#3	0.89	2.72	3.436(17)	138.5
N(2)-H(2A)...Cl(8)#3	0.89	2.58	3.328(17)	142.4
N(2)-H(2B)...Cl(1)#3	0.89	2.66	3.363(17)	136.4
N(2)-H(2B)...Cl(5)#4	0.89	2.80	3.507(16)	137.6
N(2)-H(2C)...Cl(2)	0.89	2.89	3.332(17)	112.4
N(2)-H(2C)...Cl(4)	0.89	2.45	3.289(17)	157.6
N(3)-H(3A)...Cl(2)#1	0.89	2.68	3.336(19)	131.1
N(3)-H(3A)...Cl(7)	0.89	2.71	3.366(17)	131.9
N(3)-H(3B)...Cl(1)	0.89	2.79	3.386(19)	125.7
N(3)-H(3B)...Cl(5)#5	0.89	2.65	3.423(18)	145.6
N(3)-H(3C)...Cl(3)	0.89	2.56	3.377(18)	153.2
N(4)-H(4A)...Cl(7)#6	0.89	2.50	3.354(18)	160.6
N(4)-H(4B)...Cl(2)#6	0.89	2.54	3.352(19)	152.0
N(4)-H(4B)...Cl(8)#6	0.89	2.92	3.476(17)	122.4
N(4)-H(4C)...Cl(3)#1	0.89	2.63	3.393(18)	143.6
N(4)-H(4C)...Cl(5)#5	0.89	2.81	3.435(18)	128.8

Symmetry transformations used to generate equivalent atoms:
#1 x+1/2,y-1/2,z; #2 x+1,y,z; #3 x-1/2,y+1/2,z; #4 x-1,y,z; #5 x-1/2,y-1/2,z; #6 x,y-1,z

Table S6. Hydrogen bond lengths (Å) and angles (°) for $(4\text{-ClbaH})_2CuCl_4$ at 173 K.

D-H…A	d(D-H)	d(H…A)	d(D…A)	∠(DHA)
N(1)-H(1A)...Cl(2)#4	0.89	2.93	3.576(5)	131.0
N(1)-H(1A)...Cl(4)	0.89	2.66	3.311(7)	130.6
N(1)-H(1A)...Cl(4A)#4	0.89	2.64	3.326(7)	134.8
N(1)-H(1B)...Cl(3)#5	0.89	2.59	3.373(6)	148.0
N(1)-H(1B)...Cl(3A)#4	0.89	2.65	3.334(6)	134.5
N(1)-H(1B)...Cl(4)#5	0.89	2.65	3.383(7)	140.9

N(1)-H(1B)...Cl(4A)#1	0.89	2.71	3.340(7)	128.3
N(1)-H(1C)...Cl(3)#1	0.89	2.57	3.323(6)	142.9
N(1)-H(1C)...Cl(3A)	0.89	2.57	3.291(6)	138.9
N(2)-H(2A)...Cl(3)#6	0.89	2.54	3.322(6)	146.4
N(2)-H(2A)...Cl(3A)#7	0.89	2.59	3.288(6)	136.0
N(2)-H(2B)...Cl(3)#8	0.89	2.52	3.390(6)	165.8
N(2)-H(2B)...Cl(3A)#9	0.89	2.54	3.348(6)	151.7
N(2)-H(2B)...Cl(4)#8	0.89	2.82	3.405(7)	124.9
N(2)-H(2B)...Cl(4A)#6	0.89	2.91	3.362(7)	113.3
N(2)-H(2C)...Cl(1)#7	0.89	2.96	3.530(5)	123.3
N(2)-H(2C)...Cl(4)#7	0.89	2.66	3.303(7)	129.5
N(2)-H(2C)...Cl(4A)#9	0.89	2.70	3.323(7)	128.3

Symmetry transformations used to generate equivalent atoms:

#1 x,-y,z+1/2; #4 x,y-1,z; #5 x,-y-1,z+1/2; #6 x,-y+1,z+3/2; #7 x,y+1,z+1; #8 x,-y,z+3/2; #8 x,y,z+1

Table S7. Hydrogen bond lengths (Å) and angles (°) for $(4\text{-ClbaH})_2CuCl_4$ at 298 K.

D-H…A	d(D-H)	d(H…A)	d(D…A)	∠(DHA)
N(1)-H(1A)...Cl(2)#5	0.89	2.56	3.336(7)	146.3
N(1)-H(1A)...Cl(2A)#6	0.89	2.61	3.304(7)	135.8
N(1)-H(1B)...Cl(2)#7	0.89	2.53	3.404(7)	166.0
N(1)-H(1B)...Cl(2A)#8	0.89	2.55	3.365(7)	151.8
N(1)-H(1B)...Cl(3)#7	0.89	2.83	3.414(8)	124.8
N(1)-H(1B)...Cl(3A)#5	0.89	2.92	3.372(8)	113.2
N(1)-H(1C)...Cl(3)#6	0.89	2.69	3.326(8)	129.7
N(1)-H(1C)...Cl(3A)#8	0.89	2.72	3.346(8)	128.3
N(1)-H(1C)...Cl(4)#6	0.89	2.97	3.543(6)	123.6
N(2)-H(2A)...Cl(1)#2	0.89	2.94	3.589(6)	131.3
N(2)-H(2A)...Cl(3)	0.89	2.67	3.324(8)	130.7
N(2)-H(2A)...Cl(3A)#2	0.89	2.65	3.338(8)	135.1
N(2)-H(2B)...Cl(2)#9	0.89	2.61	3.393(7)	147.9
N(2)-H(2B)...Cl(2A)#2	0.89	2.67	3.353(7)	134.3
N(2)-H(2B)...Cl(3)#9	0.89	2.67	3.408(7)	141.5
N(2)-H(2B)...Cl(3A)#3	0.89	2.73	3.365(8)	128.8
N(2)-H(2C)...Cl(2)#3	0.89	2.59	3.343(7)	142.6
N(2)-H(2C)...Cl(2A)	0.89	2.59	3.310(7)	138.8

Symmetry transformations used to generate equivalent atoms:

#1 x,-y,z-1/2; #2 x,y-1,z; #3 x,-y,z+1/2; #4 x,y+1,z; #5 x,-y+1,z+3/2; #6 x,y+1,z+1; #7 x,-y,z+3/2; #8 x,y,z+1; #9 x,-y-1,z+1/2
